# Developing a Short Assessment of Environmental Health Literacy (SA-EHL)

**DOI:** 10.3390/ijerph19042062

**Published:** 2022-02-12

**Authors:** Diana Rohlman, Molly L. Kile, Veronica L. Irvin

**Affiliations:** College of Public Health and Human Sciences, Oregon State University, Corvallis, OR 97330, USA; molly.kile@oregonstate.edu (M.L.K.); veronica.irvin@oregonstate.edu (V.L.I.)

**Keywords:** environmental health literacy, research translation, toxicology, public health

## Abstract

Environmental health literacy (EHL) is defined as the understanding of how the environment can impact human health, yet there are few tools to quantify EHL. We adapted the Short Assessment of Health Literacy (SAHL) to create the Short Assessment of Environmental Health Literacy (SA-EHL). Using the Amazon mTurk platform, users (*n* = 864) completed the 18-item SAHL and the 17-item SA-EHL. The SA-EHL was originally tested with 30 items; 13 items were removed because they were outside the acceptable difficulty parameters (DIFF: −0.4–4.0) or because of limited variance (>90% correct or incorrect), resulting in the final 17 items. Overall, participants scored highly on the SAHL, with 89.9% exhibiting high literacy. In contrast, the majority had low EHL (<1.0% high literacy, 99.2% low literacy) measured by the SA-EHL. The two scales were not correlated with each other (R^2^ = 0.013) as measured via linear regression and dichotomous variables. Scores on the SAHL and the SA-EHL were positively correlated with education. The SAHL was positively correlated with age, gender and marital status, whereas the SA-EHL was not. The SA-EHL can be used to gauge EHL for communities, and the results used to improve interventions and research translation materials.

## 1. Introduction

Environmental health literacy is most simply defined as the understanding that environmental exposures can impact health [1,2]. The discipline of environmental health incorporates public health, chemistry, environmental science, toxicology, risk communication and biology [1,2,3]. The concept of EHL follows that of health literacy: as knowledge is gained, individuals can identify relevant information, apply said information to their situation and, therefore, make informed decisions [1,2].

The conceptual framework posited for EHL uses a modified version of Bloom’s Taxonomy, organizing levels of literacy across the following categories: Recognize, Understand, Analyze, Apply, Evaluate and Create [1]. Thus, a learner recognizes important environmental health concepts, progresses to understanding them, and then uses this foundational knowledge to apply to their own situation and ultimately incorporate intervention strategies and behavior changes that mitigate the environmental hazard [1,3]. A second conceptual framework specifies three dimensions of EHL, beginning with awareness and knowledge, progressing to skills and self-efficacy, and culminating in community change [4]. Both of these frameworks are compatible and reflect individual- versus population-level changes in practices or policies that would reduce the burden of environmentally mediated disabilities or diseases.

However, there are few standardized methods to assess changes in an individual-level EHL. Evaluating individual shifts is necessary to track the adoption of environmental health knowledge within populations because increased EHL is linked with improved decision-making at both the individual and community level [3,4]. Furthermore, the ability to discern individual shifts within specific socio-demographic groups can inform the efficacy of environmental justice initiatives. With few existing tools, more qualitative measures have often been used to assess EHL [4]. Other approaches that have been used to evaluate EHL include the use of pre/post-tests, records of individual behavior changes to reduce exposure, responses to receipt of individual data (changes in awareness and behavior), and large community changes (policy and behavioral) [4]. For example, the Environmental Health Engagement Profile is a validated instrument comprising five scales looking at levels of concern and potential actions people report related to pollution [5]. Environmental media-specific tools (air, water and food) and a general EH tool were developed and validated, looking at knowledge, attitudes and behaviors related to EHL [6], and a comprehensive, 443-question instrument was developed to assess 11 core areas in environmental health [7]. Well-water specific EHL tools have recently been developed, focused on common environmental contaminants in privately owned wells in the United States [8,9].

In addition to these tools that evaluate knowledge, attitudes or skills needed for EHL, it is also important to be able to evaluate environmental health terminology [4]. EHL is built upon the existing fields of health, public health, and environmental and scientific literacy [2,3], and a tool to evaluate vocabulary that is commonly used in these fields would be useful for evaluating communications. Therefore, a simple, rapid EHL tool to assess recognition and understanding of environmental health-specific terminology is needed.

Here, we adapted the Short Assessment of Health Literacy to environmental health. The SAHL is one of the assessment tools recommended by the Agency for Healthcare Research and Quality to screen for health literacy in the clinic setting for research, practice, or program planning [10]. The SAHL is a simple test that operates on word recognition and comprehension [11]. A subject is shown a word (prompt), and then two other words. One word is correct; the other is related, but incorrect (distractor). The SAHL has been validated in English and Spanish, used in the field for over 10 years, does not require training to administer, and is quick to complete (~2–3 min) [10,11]. For example, the SAHL has been used to determine health literacy levels amongst pregnant individuals considering vaccination [12], individuals living with human immunodeficiency virus (HIV) [13] and new parents [14]. The results showed that the SAHL can be used to improve messaging and interventions based on the literacy of the target audience [12,13,14].

Words specific to environmental health were chosen to create the Short Assessment of Environmental Health Literacy (SA-EHL).

The purpose of this paper was to assess the SA-EHL using scale diagnostics and with content criterion-related and construct validity. We compared the SAHL to the SA-EHL on total scores, literacy levels and Bland–Altman plots. To evaluate construct validity, we evaluated socio-demographic variables (i.e., education, income) known to be related to health literacy [15].

## 2. Materials and Methods

We developed the SA-EHL to be in a similar format as the SAHL, a validated tool for evaluating health literacy. We developed the SA-EHL scale as per the guidelines of DeVellis [16]. In short, we: (i) generated a comprehensive list of items; (ii) reviewed items with experts; (iii) piloted the items using an online, convenience sample of adults in the United States (US); (iv) evaluated and revised items; and (v) optimized the scale. Finally, we administered the revised scale to a larger sample of adults in the US (Supplemental Information).

### 2.1. Scale Development, Item Generation

Environmental health literacy was the construct to be measured, with a focus on word recognition, and was developed to be delivered in the same manner as the SAHL. We developed the item list following a review of terms commonly used in environmental health (review of commonly used words in environmental health papers, presentations and websites), with a focus on words used to communicate research on air quality, water quality and environmental pollution. The SA-EHL was initially developed as a 30-item scale. One point was awarded for each correct response.

### 2.2. Expert Review and Inclusion of Validation Items

The SA-EHL was reviewed by experts in environmental health in the College of Public Health and Human Sciences at Oregon State University, and the Oregon Health Authority. These experts either work with programs to improve environmental health via community-engaged research or report-back on research results, science communication and public health messaging. The experts individually generated lists of items that were commonly used in their communication materials with the lay public (well water contamination, air contamination and toxic waste) and identified terms specific to environmental health. The lists were compared, and a short list was generated. The decision was made to focus on words that are foundational to the discipline, which relies on identifying and detecting hazards in environmental media, characterizing the dose or concentration of said hazards, determining how individuals are exposed and applying numerical calculations to estimate dose. For each item, our experts considered the relevance of the item to the construct, the clarity of the item to the accurate response, the appropriateness of the distractors and common use of the term in the field of environmental health.

### 2.3. Data Collection and Validation

This study used a convenience sample of adults drawn from across the U.S. via the Amazon Mechanical Turk (mTurk) online Web-based platform. This platform was used to collect socio-demographic data, and administered the SAHL and the SA-EHL. mTURK facilitates recruitment of adults (age 18+) to complete human intelligence tasks (HITS). These HITS are simple, designed to be quick and involve either human coding or interpretation of images, questions, etc. Upon successful completion of each HIT, mTurk participants receive Amazon credit. Restricting analysis to mTurk workers in the US, the sample contains a higher percentage of female, white, young and ideologically liberal individuals, relative to the general US populace [17,18,19]. However, prior research has found substantial benefits in using the mTurk platform, specifically regarding the rapid recruitment of large populations, increased compliance with study protocols and the ability to produce valid and reliable data, specifically in relationship to health literacy measures [18,19,20].

Recruitment followed a two-stage process. In the first stage, interested participants completed a pre-survey to ensure attentiveness to questions and to ensure responses were not automated. Questions included age, gender, zip code and an attention filter question. Participants who completed the pre-survey received USD 0.03 Amazon credit as per mTurk compensation guidelines. Participants who successfully completed the pre-survey were invited to the full survey via email with a unique URL. We pilot tested the protocol and revised the SA-EHL before collecting a larger sample.

A total of 166 participants piloted the survey between 24 and 27 October 2016, which included the 30-item SA-EHL scale with 2 distractors per item. A distribution of the responses for each item was reviewed. Specifically, the distractors were assessed for lack of response or majority incorrect answers, to select the most appropriate distractor. A single distractor was chosen for each item, but scale items were unchanged. Data collected from this pilot test were used to inform the scale development, but not scale validation, and are not included in this manuscript.

The revised 30-item SA-EHL was disseminated via mTurk from 21 to 25 November 2016. As previously described [8], a total of 1168 initiated the filter survey and 1153 completed the filter and were invited to the full survey. A total of 869 individuals completed the questionnaire. Full demographic information is available in Table 1.

The complete survey took approximately 20 min to complete, and compensation was offered at USD 1.00 via Amazon credit.

### 2.4. Measures

#### 2.4.1. Socio-Demographics

The following co-variates were collected: (i) age; (ii) gender; (iii) race/ethnicity; (iv) education; (v) income; and (vi) marital status. Questions followed the standardized wording from the CDC Behavioral Risk Factor Surveillance System [21].

#### 2.4.2. Short Assessment of Health Literacy (SAHL)

The SAHL is an 18-point scale, with each correct response awarded one point [11]. Higher scales are related to health literacy [11]. Each item is accompanied by one correct response, one distractor and the option “Don’t know” [11]. Table 2 contains the items, responses and distribution of responses.

#### 2.4.3. Short Assessment of Environmental Health Literacy (SA-EHL)

The Short Assessment of Environmental Health Literacy began as a 30-item scale, where each correct response is awarded one point (Table 2). Higher scores are related to higher environmental health literacy. Table 2 contains the full list of items initially selected for specificity to environmental health, along with the correct response and the distractor, and the distribution of responses.

The responses were tabulated, and items were eliminated if they met either of the following criteria: (i) >90% correct (indicating the item was too easy); (ii) <10% correct (indicating the item was too difficult); or (iii) >10% “Don’t know” (indicating the item was either too difficult or the word was unknown). Nine items meeting one or more of these criteria were eliminated due to limited variance (Table 2).

Item Response Theory Item response theory was conducted on both the SAHL and the SA-EHL. In this population, the DIFF for the SAHL fell between −4.0 and 4.0. We applied this inclusion range to the SA-EHL, thereby eliminating an additional four items. The remaining 17 items were used for all further analysis.

#### 2.4.4. Statistical Analysis

Univariate analyses and histograms were used to evaluate the distribution of responses for each scale. Scale diagnostics and psychometric properties were examined for both the SAHL and SA-EHL scales by performing a factor analysis, reviewing a scree plot and calculating the internal consistency. Factor analysis incorporated an orthogonal varimax rotation. Factors were retained with Eigenvalues greater than 1. Next, statistical tests assessed criterion-related validity between the SAHL and SA-EHL. Kappa statistics, correlations, regression coefficients and Bland–Altman plots compared the relationship between the absolute values. Ranking ability was tested by reporting the percent agreement of the two measures that fell within the same binary or tertile cutoffs. Lastly, we tested construct validity by comparing absolute scores on SA-EHL between socio-demographics (i.e., education) theoretically related to environmental health literacy. Bi-variate analyses included t-tests, chi-square and ANOVA depending on the format of the variables. All analyses were conducted using StataSE version 13 (StataCorp, College Station, TX, USA).

#### 2.4.5. Human Subjects’ Protection

As previously described [8], participants provided consent online prior to beginning the questionnaire. Consent was obtained first for the screener survey, and again for the full questionnaire. All study activities and data collection procedures were approved by the Institutional Review Board of Oregon State University (IRB protocol #7622).

## 3. Results

A full description of participant demographics was described previously [8] and is included in Table 1. A total of 911 individuals initiated the full questionnaire with 869 (95%) completing the full survey, and 864 completing both the SAHL and the SA-EHL. The sample was predominantly white (85.2%), with 6.3% reporting as Black or African American, 4.9% as Asian or Asian Indian, 2.2% as multi-racial, less than 1% as American Indian or Alaska Native, and 2% preferred to not answer or left the question blank.

### 3.1. Univariate and Scale Diagnosis

#### 3.1.1. SAHL Univariate and Scale Diagnosis

**Figure 1:**
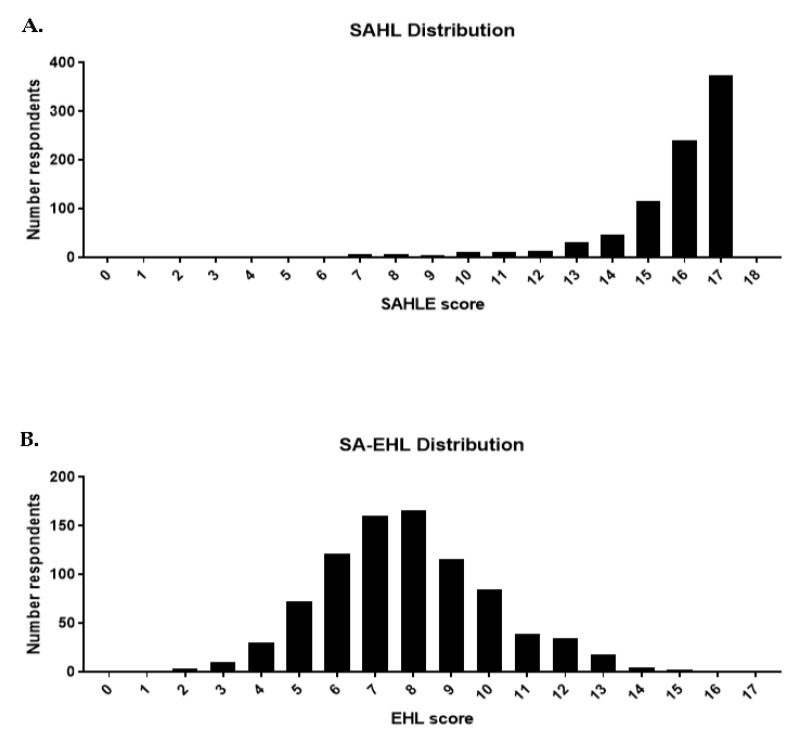
Distribution of responses per EHL scale. Distribution of SAHL (**A**) and SA-EHL (**B**) by score. The SAHL has a maximum score of 18. The SA-EHL has a maximum score of 17. *n* = 864.

Responses to the SAHL were evaluated as previously described [11]. Table 2 reports the original SAHL items and wording. Percent of correct responses for the items ranged from 2.4% to 97.5%. The mean score was 15.7 (SD = 1.90) and the median score was 16.0. A histogram is shown in Figure 1A, showing a left-skewed distribution. Cronbach’s coefficient alpha was 0.8041. A one-factor analysis was conducted, and only one factor was identified with an Eigenvalue greater than one. A scree plot (Supplemental Figure S1A) shows the drop in Eigenvalue magnitude after the first factor. Continuous scores were separated using a binary scale to delineate low (score of 0–13) and high (score of 14–18) literacy. The majority of participants had high literacy (90%) compared to low literacy (10%). To compare ranking ability between the two scales, tertiles were created for SAHLE (0–6, 7–12 and 13–18). Similarly, evaluation of the SAHL via three categories (low, medium and high) identified a majority of participants with high literacy (Table 3).

#### 3.1.2. SA-EHL Univariate and Scale Diagnosis

The full 30 items and response options are listed in Table 2. However, all scale diagnostics, criterion and construct validity analyses included only the final 17 items in the scale. The percent of correct responses for the final items (*n* = 17) ranged from 15.2% to 83.1%. The mean overall score was 7.8 (2.2 SD) and the median was 8.0. A histogram is shown in Figure 1B, showing a normal distribution. Cronbach’s coefficient alpha was 0.56. Factor analysis was conducted and a scree plot (Supplemental Figure S1B) shows the drop in Eigenvalue magnitude after the first factor, although four factors were >1 Eigenvalue. As with the SAHL, a one-factor analysis was conducted. In order to compare cutoffs with SAHLE, continuous scores were separated using a binary scale to delineate low (score of 0–13) and high (score of 14–18) literacy) and tertiles for low (0–6), medium (7–12), and high (13–17) levels of literacy.

### 3.2. Criterion-Related Validity

When looking at distribution (Figure 1A,B), we see that the distribution of scores is different between the SAHL and the SA-EHL. Therefore, we evaluated the SA-EHL via the SAHL criteria, as well as via a tertile calculation, as described in Table 3. Application of the SAHL criteria to the EHL resulted in a level of high literacy below 1% (Table 3). Evaluating the EHL categorically by tertile (low, medium and high) revealed the majority of respondents exhibited medium literacy (69.6%), with 72.5% classified as medium or high environmental health literacy. There was no agreement between the two scales using either the binary (kappa statistic = 0.0018, 10.8% agreement and *p* = 0.18) or the tertile method (kappa statistic = 0.005, 7.6% agreement and *p* = 0.14).

**Figure 2:**
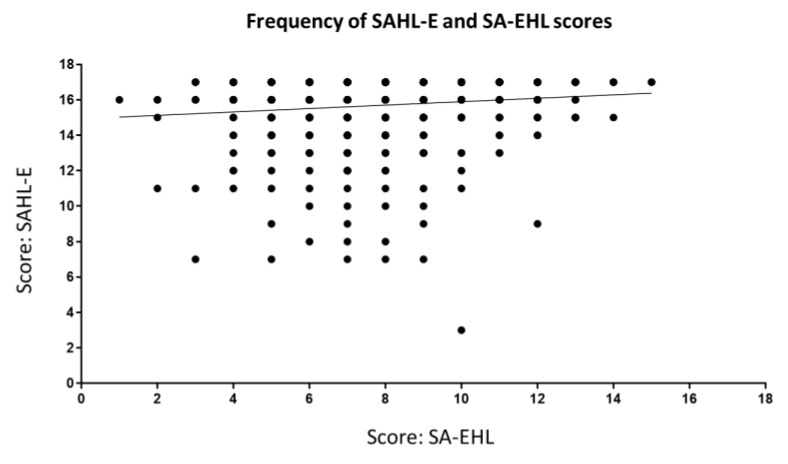
Distribution of SAHL and SA- EHL scores. Absolute scores for the SAHL (max = 18) and the SA-EHL (max = 17) were plotted. A linear regression revealed poor correlation between the two scores (r^2^ = 0.013).

**Figure 3:**
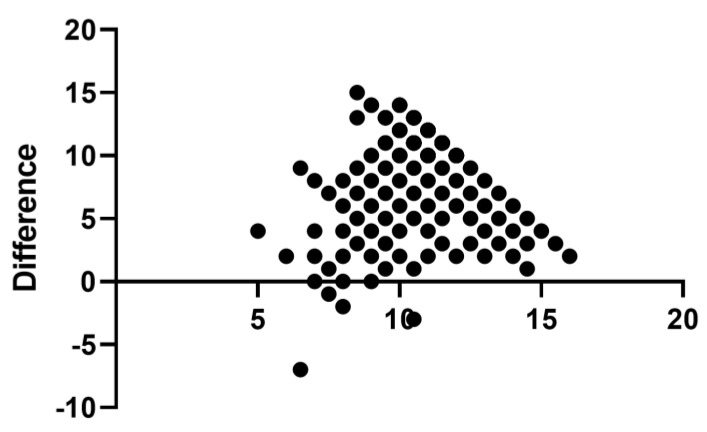
Bland–Altman plot depicting the difference between the SAHL and the SAEHL versus the average.

The two scales were poorly correlated with each other. A simple linear regression revealed no correlation (R^2^ = 0.013, *p* < 0.001) (Figure 2). A Bland–Altman analysis was conducted (Figure 3) and did not reveal any pattern of agreement between the two scales. A low correlation was observed between the SAHL and the SA-EHL (correction of r = 0.11, *p* < 0.001). When the SAHLE was regressed on EHL_17, it produced a small but significant regression coefficient of 0.13 (standard error = 0.04, 95% CI 0.05, 0.21, and *p* < 0.001). The model fit was low but significant (not shown in table—model statistics F (1, 862) = 11.12, model *p* < 0.001, R^2^ = 0.012). Even though the model produced significant correlations, the magnitude was low and not meaningful. The mean difference between the scores of the two scales was 7.85 (2.8 SD).

Individual items within the SAHL and the SA-EHL were evaluated using linear regressions. Across all items, the R^2^ value was ≤0.3, indicating little to no correlation between any items on the SAHL and the SA-EHL.

### 3.3. Construct Validity

Construct validity was determined using a bivariate analysis between the absolute score and hypothetically related variables for each scale. We evaluated known predictors or characteristics of health literacy, including age, gender, income, education and marital status. Race/ethnicity was not assessed as the respondent population was predominantly White (77.7%, Table 1). Four characteristics were positively correlated with the SAHL: age (*p* < 0.001); gender (*p* < 0.001); education (*p* < 0.001); and marital status (*p* < 0.001) (Table 1). None of the other co-variates were correlated with the SAHL.

For the SA-EHL, only education was positively related to the absolute score for the SA-EHL (*p* < 0.001), where individuals with a college degree had the highest scores within the SA-EHL. All other co-variates were not correlated with the SA-EHL (Table 1).

## 4. Discussion

We developed and assessed the Short Assessment of Environmental Health Literacy (SA-EHL) tool. This scale was adapted from the Short Assessment of Health Literacy [11]. Our tool performed differently from the SAHL, as assessed by the distribution of responses and poor scale correlation. In contrast, our prior research with the WELLS tool identified moderate correlations between health literacy and EHL scales [8]. However, the WELLS tool relied heavily on numeracy, which may explain the correlation between the scales [8]. In contrast, the SAHL and SA-EHL relies on word recognition. Here, our sampled population demonstrated very high health literacy via the SAHL, yet moderate-to-poor environmental health literacy using the SA-EHL. In fact, the mean score for health literacy was twice as high as the mean score for environmental health literacy. This may be due to either conflicting definitions of terms, or unfamiliarity with environmental health terminology. A review of EHL found that a common barrier was the use of confusing or unknown terms [4], either due to low literacy or cultural context [3,22]. Of note, items with very poor recognition (>90% incorrect or >90% “Don’t know”) were removed from our SA-EHL to mitigate this issue. However, many of the terms surveyed have varying definitions based on popular versus scientific use. Science communication experts have advocated that such dual-use terms be avoided to aid in comprehension [23]. Finally, the reliance on word recognition may also contribute to the poor correlation, as people may be more familiar with health-related terms, and less familiar with environmental health terms. This recognition does not always translate to true literacy, with evidence that word recognition assessments may overestimate health literacy [24]. Ultimately, our tool indicates that competence in health literacy does not directly translate to environmental health literacy. We did find that EHL was associated with educational attainment. This may be a function of increased education leading to higher literacy and, therefore, stronger word recognition and comprehension. This was identified previously with the WELLS scale being correlated with both income and education [8].

Recently, a survey of environmental health researchers, clinicians, educators and community partners was conducted to identify essential competencies in EHL [25]. Respondents were asked to rank the knowledge of specific environmental health terms. While the term “hazard” was ranked as essential by 67.2%, other terms were viewed as less important: “dose” (57.5%); “risk” (57.9%); and “frequency of exposure” (56.6%) [25]. Rather, the panel focused on specific skills they felt were essential for EHL, such as ability to find information to reduce risks in their life, or ability to identify established hazards in their environment [25]. Our results indicate that basic knowledge of frequently used terms in environmental health may be needed before individuals can build these higher-level competencies.

The SA-EHL is designed to assess the lower levels of EHL, comprising recognition and knowledge, or the dimension of awareness and understanding [1,4]. Additional tools will be needed to assess higher-level competencies. However, this tool can be used to generate a standardized, baseline level of EHL founded on recognition and word familiarity. Once this baseline is set, additional interventions or education can be conducted to scaffold individuals and communities to other important concepts in EHL [9]. Additionally, this baseline can help researchers and public health professionals better tailor messaging around environmental health hazards. Specifically, we envision this tool, among others, to be a useful way to ensure materials match the EHL of the target audience. By gauging baseline EHL through the use of focus groups or community forums, researchers and practitioners can avoid developing complex or repetitive materials. This is the theory behind health literacy screeners, which are often used to adjust the literacy level of materials, or the format in which they are disseminated (e.g., use of pictorial descriptions instead of text) [26,27]. In the same way, the SA-EHL may be used as an EHL screener to inform more appropriate messaging. This is a priority especially given environmental hazards following disasters, including wildfires, hurricanes and chemical releases [28,29,30].

We acknowledge several limitations with this study. The SA-EHL scale reflects words that were selected by public health and environmental health researchers. When compared to the aforementioned survey, several of these terms are considered essential, such as “dose”, “exposure” and “risk” [25], yet the final SA-EHL scale is not representative of the full range of environmental health. Furthermore, the test population was highly educated and not fully representative of the United States [17,18,19]. This highlights an important issue for environmental health educators, which is the inclusion of definitions for common terms that are considered scientifically essential but may be misunderstood by a more lay audience. The results reported herein may not be applicable to other countries with differing cultural, linguistic and socioeconomic contexts. Countries with different pollution burdens may also differ in baseline awareness of environmental health concerns. It is interesting that the SA-EHL was correlated strongly with education, and not with additional factors, whereas the SAHL was correlated with multiple factors. This, along with the additional limitations described, suggests that this preliminary research be a foundation for additional work testing the SA-EHL in diverse populations. Finally, there remains a question as to what the test is fully measuring—environmental health literacy, or familiarity with words?

When evaluating word difficulty, Lee and Kauchak found that words with a lower frequency of occurrence in the English language had a higher level of perceived difficulty and were less frequently correctly defined [31]. In contrast, more frequent words were perceived as easier, and more correctly defined. Interestingly, there was no correlation between word length and ability to define the word [31], although longer words were perceived as more difficult [31].

There remain many strengths with this study. Using mTurk, the tool was tested in over 800 individuals across the United States. We adapted a validated health literacy tool, the Short Assessment of Health Literacy, allowing for an analysis of the overlap between health literacy and EHL. However, replications and further testing of this new measure of environmental health literacy should be considered.

## 5. Conclusions

The Short Assessment of Environmental Health Literacy was developed to create a standardized tool capable of assessing an individual’s awareness (recognition) and knowledge of environmental health-specific terminology. While focused on word recognition, this tool can be valuable to gauge the baseline EHL of a population, thereby enabling researchers and public health practitioners to tailor educational materials [22]. This may mitigate the existing EHL barrier of confusing or unknown terminology [4]. Additionally, studies that are returning data to study participants and communities have reported qualitative increases in EHL with the return of data [32,33,34,35]. Thus, there is a need for standardized scales that can inform researchers and health care professionals about baseline EHL levels [5,6,36] and assess changes in EHL upon receipt of data [3]. The SA-EHL is designed to be a short, rapid assessment of baseline knowledge related to environmental health terms and concepts. The data from the SA-EHL can be used to measure changes in EHL and to tailor educational or outreach materials to the language of the target audience.

## Figures and Tables

**Table 1:** Study population demographics. Covariates included age, gender, educational attainment and household income.

Characteristic	Univariate Distribution	SAHL ^a^	SA-EHL ^b^
Total sample	*n* = 864 ^c^		
	Mean (SD)	Correlation	Correlation
Age *	38 (11.6)	0.16 (*p* < 0.01)	0.03 (*p* = 0.35)
	Percentage (*n*)	Mean (SD)	Mean (SD)
Gender *		*p* < 0.01	*p* = 0.9
Male	41.3% (357)	15.4 (2.1)	7.9 (2.1)
Female	57.9% (500)	15.9 (1.7)	7.8 (2.3)
Prefer not to answer	<1% (5)	15.6 (1.7)	7.4 (1.8)
Missing	<1% (2)	15.5 (2.1)	7.5 (0.7)
Ethnicity			
Hispanic or Latino	6.8% (59)	15.3 (2.2)	7.6 (2.3)
Not Hispanic or Latino	93.1% (804)	15.7 (1.9)	7.9 (2.2)
Prefer not to answer	<1% (1)	14 (0)	7 (0)
Missing	0% (0)		---
Education *		*p* ≤ 0.001	*p* ≤ 0.001
College Graduate	52% (449)	15.7 (1.8)	8.1 (2.3)
Some College	34.5% (298)	15.8 (1.8)	7.7 (2.1)
High School Graduate	12.4% (107)	15.4 (2.2)	7.3 (2.2)
Some High School	<1% (2)	13.5 (4.9)	6 (4.2)
Prefer not to answer	<1% (6)	12 (3.5)	7 (2.9)
Missing	<1% (2)	15 (1.8)	8.5 (1.3)
Income		*p* = 0.36	*p* = 0.26
USD 0–35,999	35.2% (304)	15.6 (2.1)	7.4 (2.2)
USD 36,000–50,000	19.2% (166)	15.5 (1.9)	7.7 (2.4)
USD 51,000–75,999	19.4% (168)	15.9 (1.6)	7.8 (2.3)
USD 76,000 or higher	24.0% (207)	15.7 (1.7)	8.1 (2.1)
Prefer not to answer	2.2% (19)	15.6 (2.4)	7.4 (2.0)
Missing	0% (0)	---	---
Rent/own		*p* = 0.7	*p* = 0.6
Own	49.2% (425)	15.8 (1.8)	7.9 (2.2)
Rent	43.9% (379)	15.6 (2.1)	7.7 (2.3)
Other arrangement	6.8% (59)	15.8 (1.8)	7.8 (2.0)
Missing	<1% (1)	15 (0)	9 (0)
Marital Status *		*p* ≤ 0.0001	*p* = 0.72
Married	44.5% (383)	15.6 (2.0)	7.8 (2.2)
Divorced	8.6% (74)	16.2 (1.1)	7.9 (2.3)
Widowed	1.4% (12)	15.8 (2.1)	7.8 (1.3)
Separated	1.5% (13)	16.7 (0.5)	8.2 (2.0)
Never married	28.6% (247)	15.4 (2.1)	7.9 (2.2)
Unmarried couple	14.2% (123)	16.1 (1.3)	7.8 (2.4)
Prefer not to answer	1.0% (9)	13.2 (3.8)	6.7 (2.5)
Missing	<1.0% (3)	---	---

^a^ Numbers are mean score and standard deviation (SD) between each demographic or household variable and the ^a^ SAHL (Short Assessment of Health Literacy) or the ^b^ SA-EHL (Short Assessment of Environmental Health Literacy. Calculations were performed with either *t*-tests or ANOVAs. ^c^ Total sample size is 864. Numbers are sample size and percentage for each variable. * Indicates that there is a significant difference in scores between demographic or household characteristics. *p* less than 0.05.

**Table 2:** List of SAHL and SA-EHL items (left column) and possible responses (correct response, distractor, “Don’t know”/skipped).

SAHL-E		*n*	%	DIFF
Dose	Amount	843	97.6	−3.28
	Sleep	20	2.3	
	Don’t know/skipped	1	<1.0	
Kidney	Urine	797	92.3	−3.10
	Fever	24	2.8	
	Don’t know/skipped	43	5.0	
Medication	Treatment	832	96.3	−3.0
	Instrument	29	3.4	
	Don’t know/skipped	3	<1.0	
Miscarriage	Loss	847	98.0	−3.87
	Marriage	9	1.0	
	Don’t know/skipped	8	<1.0	
Alcoholism	Addiction	814	94.2	−2.60
	Recreation	45	5.2	
	Don’t know/skipped	5	<1.0	
Hormones	Growth	792	91.7	−2.65
	Harmony	42	4.9	
	Don’t know/skipped	30	3.5	
Pregnancy	Birth	823	95.3	−2.95
	Childhood	29	3.4	
	Don’t know/skipped	12	1.4	
Seizure	Dizzy	781	90.4	−3.14
	Calm	23	2.7	
	Don’t know/skipped	60	6.9	
Abnormal	Different	834	96.5	−3.10
	Similar	25	2.9	
	Don’t know/skipped	5	<1.0	
Nerves	Anxiety	838	97.0	−3.24
	Bored	21	2.4	
	Don’t know/skipped	5	<1.0	
Constipation	Blocked	814	94.2	−2.64
	Loose	43	5.0	
	Don’t know/skipped	7	<1.0	
Hemorrhoid	Veins	705	81.6	−2.64
	Heart	38	4.4	
	Don’t know/skipped	121	14	
Syphilis	Condom	573	66.3	−1.12
	Contraceptive	173	20.0	
	Don’t know/skipped	118	13.7	
Directed	Instruction	750	86.8	−1.85
	Decision	99	11.5	
	Don’t know/skipped	15	1.7	
Occupation	Work	833	96.4	−3.03
	Education	27	3.1	
	Don’t know/skipped	4	<1.0	
Nutrition	Healthy	838	97.0	−3.51
	Soda	15	1.7	
	Don’t know/skipped	11	1.3	
Infection	Plant	21	2.4	3.14
	Virus	833	96.4	
	Don’t know/skipped	10	1.2	
Diagnosis	Evaluation	818	94.7	−2.70
	Recovery	40	4.6	
	Don’t know/skipped	6	<1.0	
SA-EHL		*n*	%	DIFF
Response ^a^	Endpoint	36	4.2	---
	Answer	809	93.6	
	Don’t know/skipped	19	2.2	
Exposure ^a^	Contact	819	94.8	---
	Consumed	32	3.7	
	Don’t know/skipped	13	1.5	
Concentration	Amount	651	75.4	−2.69
	Strong	206	23.8	
	Don’t know/skipped	7	0.8	
Chemical	Substance	637	73.7	−2.56
	Solution	214	24.8	
	Don’t know/skipped	13	1.5	
Acute	Short	251	29.1	2.00
	Sharp	588	68.1	
	Don’t know/skipped	25	2.9	
Chronic	Long	649	75.1	−2.70
	Disease	205	23.7	
	Don’t know/skipped	10	1.2	
Risk	Possibility	192	22.2	2.92
	Hazard	670	77.6	
	Don’t know/skipped	2	0.2	
Particulate ^a^	Dust	553	64.0	---
	Sand	211	24.4	
	Don’t know/skipped	100	11.6	
Aerosol	Particle	131	15.2	3.96
	Spray	717	83.0	
	Don’t know/skipped	16	1.9	
Background	Natural	192	22.1	2.65
	Explanation	589	68.2	
	Don’t know/skipped	84	9.7	
Fraction	Amount	467	54.1	−0.43
	Division	389	45.0	
	Don’t know/skipped	8	0.9	
Media	Environment	227	26.3	2.13
	Digital	566	65.5	
	Don’t know/skipped	71	8.2	
Organic	Carbon	302	35.0	1.33
	Vegetables	533	61.7	
	Don’t know/skipped	29	3.4	
Remediate ^a^	Clean	214	24.8	---
	Repair	533	61.7	
	Don’t know/skipped	117	13.5	
Susceptibility	Risk	190	22.0	2.94
	Vulnerable	665	77.0	
	Don’t know/skipped	9	1.0	
Safe	Secure	570	66.0	−1.68
	Clean	280	32.4	
	Don’t know/skipped	14	1.6	
Treatment ^b^	Fix	715	82.8	−4.04
	Drug	127	14.7	
	Don’t know/skipped	22	2.6	
Monitor ^b^	Watch	767	88.8	−4.84
	Investigate	96	11.1	
	Don’t know/skipped	1	0.1	
Contaminant ^b^	Pollutant	751	86.9	−4.56
	Not belong	106	12.3	
	Don’t know/skipped	7	0.8	
Level	Amount	711	82.3	−3.75
	Even	143	16.6	
	Don’t know/skipped	10	1.2	
Test	Measure	575	66.6	−1.64
	Exam	286	33.1	
	Don’t know/skipped	3	0.4	
Source	Origin	718	83.1	−3.82
	Reference	140	16.2	
	Don’t know/skipped	6	0.7	
Uncertainty ^b^	Range	112	13.0	4.41
	Unsure	743	86.0	
	Don’t know/skipped	9	1.0	
Artifact ^a^	Effect	170	19.7	---
	Remainder	565	65.4	
	Don’t know/skipped	129	14.9	
Manipulation	Process	149	17.3	3.54
	Change	682	78.9	
	Don’t know/skipped	33	3.8	
Error	Difference	159	18.4	3.46
	Mistake	698	80.8	
	Don’t know/skipped	7	0.8	
Threshold ^a^	Concentration	63	7.3	---
	Limit	783	90.6	
	Don’t know/skipped	18	2.1	
Variability ^a^	Range	795	92.0	---
	Error	64	7.4	
	Don’t know/skipped	5	0.6	
Effects ^b^	Change	759	87.9	−6.00
	Move	57	6.6	
	Don’t know/skipped	48	5.6	
Chance ^a^	Accidental	779	90.2	---
	Probability	82	9.5	
	Don’t know/skipped	3	0.4	

^a^ Items were removed from the scale if responses were >90% correct, <10% correct or >10% “Don’t know”. ^b^ Items were removed if the diff was outside a −4.0–4.0 range.

**Table 3:** Description of EHL levels and frequency of participant responses.

Variable	Health Literacy (SAHL) Title 3		Environmental Health Literacy (SA-EHL)	
	Scale Range	% Respondents	Scale Range	% Respondents
SAHL Criteria ^1^				
Low	0–13	10.1	0–13	99.2
High	14–18	89.9	14–17	0.8
Tertile criteria ^2^				
Low	0–6	0.1	0–6	27.6
Medium	7–12	6.4	7–12	69.6
High	13–18	93.5	13–17	2.9

^1^ The SAHL method uses a binary scale where scores from 0 to 13 are considered low literacy and scores 14 to 18 are considered high literacy. Agreement between the two measures dichotomized was low with kappa statistic = 0.0018, 10.8% agreement and *p* = 0.18. ^2^ The SA-EHL scale was divided into tertiles (0–6, 7–12 and 13–17) and applied to the responses. Agreement between the two measures as tertiles was low with kappa statistic = 0.005, 7.6% agreement and *p* = 0.14.

## Data Availability

The data presented in this study are available on request from the corresponding author.

## Supplementary Materials

The following are available online at https://www.mdpi.com/article/10.3390/ijerph19042062/s1, Figure S1: Scree plot for the SAHL (A) and the SA-EHL (B). Short Assessment of Environmental Health Literacy.
