# Supplementary figures and images for: Developing a Short Assessment of Environmental Health Literacy (SA-EHL)
Source: Int J Environ Res Public Health. 2022 Feb 12;19(4):2062. doi: 10.3390/ijerph19042062 (PMC8872614; doi:10.3390/ijerph19042062)

A.

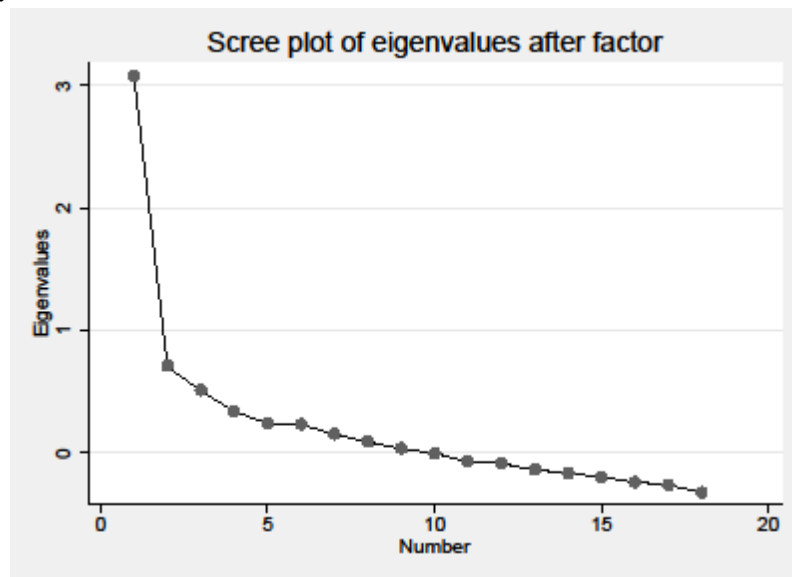

B.

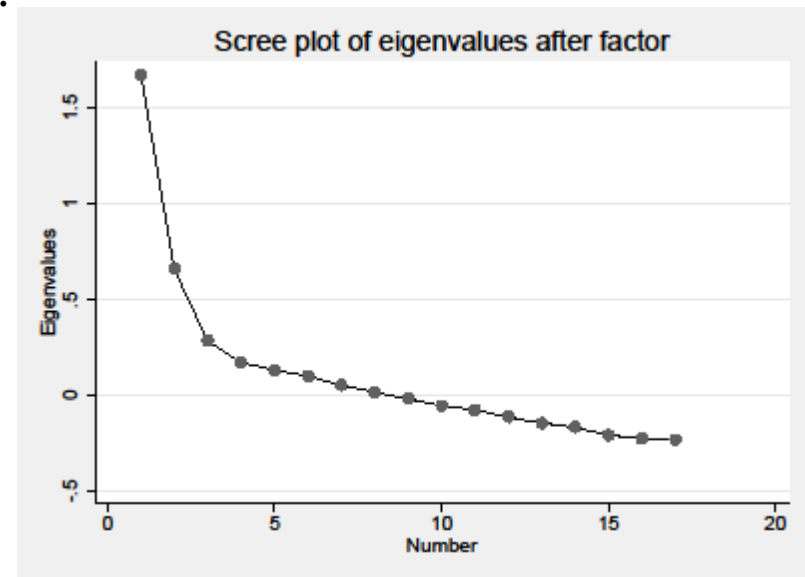

Supplemental Figure S1. Scree plot for the SAHL (A) and the SA-EHL (B).

Supplement: Supplementary file 1 [file ijerph-19-02062-s001.zip › ijerph-1534344-supplementary.pdf]
